# Obesity and the Gut-Brain Axis in Type 1 Diabetes Mellitus: Terra Incognita?

**DOI:** 10.1007/s13679-025-00654-8

**Published:** 2025-07-25

**Authors:** Georgia Argyrakopoulou, Evdoxia Gitsi, Maria Dalamaga, Alexander Kokkinos

**Affiliations:** 1 Diabetes and Obesity Unit, Athens Medical Center, Athens, 15125 Greece; 2 Department of Biological Chemistry, Medical School, National and Kapodistrian University of Athens, Athens, 11527 Greece; 3 First Department of Propaedeutic Internal Medicine, Medical School, National and Kapodistrian University of Athens, Laiko General Hospital, Athens, Greece

**Keywords:** Bariatric surgery, Glucagon-like peptide-1, GLP-1, Gut-brain axis, Insulin resistance, Obesity, T1DM, Type 1 diabetes mellitus

## Abstract

**Purpose of Review:**

The increasing prevalence of obesity among individuals with type 1 diabetes mellitus (T1DM) presents a significant clinical challenge, as it exacerbates insulin resistance, impairs glycemic control, and increases cardiometabolic risk. While obesity in T1DM is influenced by both genetic and environmental factors, recent evidence highlights the role of the gut-brain axis in metabolic regulation. This review explores the complex relationship between obesity and T1DM, the role of the gut-brain axis in metabolic dysregulation, and current weight management strategies, highlighting the need for further research to optimize treatment outcomes in this unique patient population.

**Recent Findings:**

Key gastrointestinal hormones, including glucagon-like peptide-1 (GLP-1), glucose-dependent insulinotropic peptide (GIP), and amylin, play essential roles in appetite control, energy balance, and glucose metabolism, yet their dysregulation in T1DM remains poorly understood. Addressing obesity requires a multifaceted approach, including lifestyle modifications, pharmacotherapy with GLP-1 receptor agonists (GLP-1RAs), and bariatric surgery (BS). Although limited, accumulating evidence regarding the use of liraglutide, semaglutide and tirzepatide in T1DM begin to highlight the safety and effectiveness of these molecules in this subset of patients as well.

**Summary:**

Lifestyle modifications, GLP-1 RAs based pharmacotherapy and BS have emerged as potential strategies to address obesity in patients with T1DM. Initial findings point to potential improvements in both metabolic health and glycemic control, but further exploration of their role in the co-occurrence of obesity and T1DM remains limited. Ongoing research is crucial to better understand how the gut-brain axis influences weight regulation in T1DM and to determine the sustained benefits and risks of these emerging therapies.

## Introduction

According to the latest global data, an estimated 8.4 million individuals worldwide were living with type 1 diabetes mellitus (T1DM) in 2021. Among them, 18% were under 20 years old, 64% were aged 20–59 years, and 19% were aged 60 years or older. By 2040, the prevalence of T1DM is projected to rise significantly, reaching 13.5–17.4 million cases—a 60 to 107% increase compared to 2021—with the largest relative increase anticipated in low-income and lower-middle-income countries [1].

Alongside the growing incidence of T1DM, obesity rates among individuals with this condition have also been rising, with prevalence estimates ranging between 2.8% and 37.1% over recent decades [2, 3]. This rise is of particular interest because it appears to be associated with an increased cardiometabolic risk and a heightened likelihood of developing chronic complications compared to lean counterparts of these patients. Furthermore, insulin resistance, which frequently accompanies obesity, exacerbates the challenge of glycemic control and weight management by increasing insulin requirements [3].

Similar to the general population, obesity in T1DM is influenced by multiple factors, including non-modifiable endogenous elements such as genetic predisposition, epigenetics, gut hormone profiles, and intestinal microbiota. Additionally, modifiable exogenous factors, including altered eating behaviors, eating disorders, sedentary time, short sleep duration, chronic stress, psychosocial influences, and unhealthy lifestyle habits, also contribute to its development [4]. Importantly, insulin therapy is no longer considered the primary driver of obesity in T1DM, as suggested by current evidence [3]. It does, however, contribute to obesity in conjunction to other factors such as those mentioned above. In terms of T1DM, one additional significant concern is the fear of hypoglycemia that frequently leads to excess energy consumption. Increased age at onset, longer duration of the disease, and a diagnosis after puberty appear to be associated with a higher co-existence of obesity and T1DM [5]. A meta-analysis of 9 studies with 2,658 cases found evidence linking childhood obesity or higher body mass index (BMI) to a higher risk of T1DM [6].

Over the past few decades, substantial evidence has been gathered indicating that the gut-brain axis plays a crucial role in regulating energy balance and food intake. Key gastrointestinal hormones, such as ghrelin, cholecystokinin, glucose-dependent insulinotropic polypeptide (GIP), glucagon-like peptide-1 (GLP-1), and others, act as mediators between the gut and the brain. In individuals with T1DM, hormonal dysregulation—particularly of insulin, amylin (which is co-secreted with insulin, delays gastric emptying and suppresses appetite), and glucagon—has been documented, yet the underlying mechanisms remain poorly understood [7, 8]. This review aims to fill that knowledge gap and explore the interplay between obesity and T1DM, available data on the role of the gut-brain axis, as well as discuss the most prevalent current interventions for weight management in patients with a BMI ≥ 27 kg/m^2^, including GLP-1 receptor agonist (GLP-1RA) therapy and bariatric surgery (BS).

### The Role of the Gut Brain Axis in the Co-Existence of Obesity and T1DM

Few studies have examined the gut-brain axis in T1DM, focusing primarily on incretin secretion and its link to glycemia and gastric emptying. It is well known that fasting plasma ghrelin concentrations are negatively correlated with BMI, and its levels decrease postprandially. In obesity, the postprandial decline in plasma ghrelin and leptin is blunted compared to lean counterparts, resulting in greater food intake [9]. Ghrelin concentrations in normal weight patients with newly diagnosed T1DM prior to the initiation of insulin therapy have been found reduced. Furthermore, following insulin treatment, ghrelin levels were not suppressed during a mixed meal test (33% fat, 50% carbohydrates and 17% proteins). It could be presumed that ghrelin’s low concentrations at diagnosis could be due to insulin deficiency, hyperglycemia, and disrupted lipid metabolism [10].

The secretion of incretins is influenced by the size and nutritional constituents of a meal [11]. Meal challenges in T1DM patients have shown significant postprandial increases in GLP-1 and GIP concentrations. While several studies have shown a reduced incretin effect in patients with T2DM, incremental GLP-1 and GIP responses were normal in T1DM compared to healthy controls [12, 13].

Another noteworthy observation has been conducted in patients with residual beta cell secretion, that may persist even in long-standing diabetes. A study involving 25 patients with T1DM was conducted using a mixed meal tolerance test in order to investigate whether residual insulin secretion may influence glucagon and GLP-1 responses. No influence of residual beta cell function C-peptide secretion on peak glucagon levels response was identified. However, a significant correlation between GLP-1 and glucagon secretion was identified in C-peptide-micro-insulin secretion individuals (patients that still secrete minute insulin quantities) and patients with recent-onset diabetes. Interestingly, an inverse correlation between GLP-1 and C-peptide levels was also noted. The authors point out the possibility that the increased GLP-1 secretion in C-peptide-negative participants could be linked to reduced nutrient absorption related to pancreatic exocrine deficiency and increased delivery of nutrients and/or fermentation products to distal intestinal L cells [14].

The comparison of the postprandial responses of GIP, GLP-1, ghrelin and Insulin-Like Growth Factor Binding Protein-1 (IGFBP-1) in a high- vs. low-fat meal has also been investigated in a small number of adolescents with T1DM. Notably, while the given amount of insulin was the same, the total energy content was different between the two meals (greater in the high-fat meal). The postprandial levels of GIP and GLP-1 were significantly elevated while ghrelin was reduced following a meal with higher fat and energy content. Specifically, the peak postprandial 4-hour response of GIP was markedly larger and the 2-hour response of GLP-1 was larger after a meal with high fat and energy content. The ghrelin response was also larger after the high-fat meal, while the IGFBP-1 response did not differ between the high- and low-fat meals, probably because the same prandial insulin dose was given at both meals [15].

GLP-1 is known to enhance insulin secretion and inhibit glucagon secretion [11]. Experiments using intravenous infusion of GLP-1 in patients with T1DM reduced glucose levels and inhibited glucagon secretion, partly because of GLP-1 augmented insulin sensitivity as well as due to the effect of GLP-1 on gastric emptying [11], the latter playing a role primarily in insulin-deficient T1DM patients [16].

### Therapeutical Approaches

The management of obesity in individuals with T1DM presents unique challenges that require a multifaceted approach. While traditional methods such as diet, exercise, and behavioral interventions are central to treatment, these are not sufficient for patients with T1DM living with obesity. Insulin resistance, impaired glucose control, and the risk of hypoglycemia complicate weight management in this population.

In fact, patients with T1DM have often been excluded from clinical trials evaluating anti-obesity medications, leaving a gap in the evidence supporting their use. Nevertheless, a growing body of research is investigating pharmacotherapy as an adjunct to lifestyle changes for treating obesity in T1DM (Table 1). Among the current popular pharmacotherapies for glucose control, comorbidity management, and obesity in adults with T1DM, GLP-1 and dual GLP-1/GIP receptor agonists may offer promising benefits, particularly in improving insulin sensitivity and aiding in weight management, though their long-term effects in T1DM still require further investigation [3].

**Table 1:** Gut hormones in T1DM & obesity

Hormone	General Action	Action in Obesity	Action in T1DM
Ghrelin	“Hunger hormone” – increases during fasting, decreases postprandially	Blunted postprandial decline – contributes to increased food intake	Low levels at diagnosis before initiation of insulin therapy. After insulin initiation, not adequately suppressed postprandially.
GLP-1	Enhances insulin secretion, reduces appetite & food intake, slows gastric emptying	Reduced incretin effect	Normal postprandial response. In C-peptide-negative patients: increased GLP-1 secretion. Intravenous GLP-1 reduces glucose and glucagon levels.
GIP	Stimulates insulin secretion, reduces appetite & food intake	Ambiguous role in appetite, potent incretin effect	Normal postprandial response. Increased response after high-fat meals.
Glucagon	Increases blood glucose via glycogenolysis and gluconeogenesis	Elevated levels but possible resistance	Unaffected by residual insulin secretion. Correlation observed between GLP-1 and glucagon in patients with minimal C-peptide secretion.
IGFBP-1	Regulates IGF-1 bioavailability – suppressed by insulin	Variable; may be lower in insulin-resistant states	Postprandial response did not differ between high- and low-fat meals (same insulin dose used).

GLP-1 = Glucagon-Like Peptide-1, GIP = Gastric Inhibitory Polypeptide, IGFBP-1 = Insulin-Like Growth Factor Binding Protein-1

#### Exenatide

Exenatide was the first GLP-1RA to receive approval from the Food and Drug Administration (FDA) in 2005 [17]. The safety and effectiveness of exenatide have been evaluated in individuals with T1DM, but mainly without obesity. Studies have shown that exenatide can reduce postprandial glucose excursions and modestly decrease HbA1c levels. Additionally, it has been associated with small weight loss and lower daily insulin requirements without increasing hypoglycemia risk [17]. The most frequently reported side effects were nausea and vomiting, consistent with the known adverse reactions associated with GLP-1RAs [17].

A network meta-analysis comprising 23 randomized controlled trials (*n* = 5,151 T1DM patients) with an average study duration of 30.8 ± 14.5 weeks—including participants with overweight and obesity—revealed that the insulin + exenatide group experienced a significant reduction in body weight of 5.1 kg compared to the insulin-only group. However, no notable changes were observed in HbA1c levels, daily insulin dose, or hypoglycemic episodes relative to baseline [18].

#### Liraglutide

Liraglutide is the most studied GLP-1RA for its ability to manage obesity and improve glycemic control in T1DM patients. The Lira-1 trial was a landmark study assessing the safety and efficacy of liraglutide (1.8 mg) added to insulin therapy in adults with T1DM and BMI > 25 kg/m^2^ [19]. This randomized, double-blind, placebo-controlled trial found that liraglutide reduced body weight by 6.8 kg compared to the placebo group, with a 5.8 IU reduction in daily insulin doses. Although there was no significant change in HbA1c between the liraglutide and placebo groups, the reductions in insulin requirements and body weight suggest a potentially beneficial effect on insulin sensitivity. Importantly, liraglutide was associated with a reduced incidence of hypoglycemic events, suggesting it could help mitigate the risk of hypoglycemia in this population [19]. This reduction in hypoglycemic episodes is particularly notable, given that insulin-treated T1DM patients are at high risk for these events, especially during weight loss interventions that typically require insulin dose reductions [19].

The ADJUNCT ONE and TWO trials found similar results in terms of body weight and insulin reduction with the 1.8 mg dose of liraglutide, but with an additional modest decrease in HbA1c, as well as an increase in hypoglycemia and hyperglycemia with ketosis. In this series of studies, around 1400 patients with T1DM were randomized to receive liraglutide at doses of 1.8 mg, 1.2 mg, or 0.6 mg daily for 52 weeks. In the group receiving the highest dose (1.8 mg), patients experienced a mean weight loss of 4.9 kg, along with an 8% reduction in total insulin dose, suggesting enhanced insulin sensitivity [20]. While liraglutide was generally well tolerated, with the exception of some mild gastrointestinal side effects (mainly nausea and diarrhea), the drug was linked with a higher incidence of mild hypoglycemia, especially in the higher-dose groups. The occurrence of hypoglycemia was a common concern in these studies, which aligns with the clinical need to carefully adjust insulin doses when initiating GLP-1RAs. This is partially because of the appetite-suppressing effects of liraglutide leading to lower food intake, potentially increasing the risk of hypoglycemia, especially when insulin doses are not properly down titrated. Additionally, there was an increased rate of hyperglycemia with ketosis in some patients, particularly those who reduced their insulin doses too aggressively. This highlights the need for careful monitoring and gradual adjustments in both insulin therapy and liraglutide dosing to avoid such complications, as well as the conduction of further studies with more flexible and individualized approaches [20].

In a separate study of 72 patients with T1DM and overweight or obesity, liraglutide (1.2 mg and 1.8 mg) over 12 weeks resulted in modest reductions in glucose levels, HbA1c, and significant weight loss (5 ± 1 kg), along with small reductions in total insulin dose (-10-12 IU). Gastrointestinal side effects, especially nausea, were more frequent, but hypoglycemia incidence did not increase. As expected, no significant changes in C-peptide levels were observed, likely due to the baseline undetectable C-peptide levels in the participants [21]. A retrospective study in a sample with the same conditions further supported these findings, showing reductions in mean glucose levels, HbA1c, and body weight, along with significant decreases in total daily and bolus insulin doses (*p* = 0.008 and *p* = 0.011, respectively), without an increase in hypoglycemia [22].

The Lira Pump trial evaluated liraglutide (1.8 mg daily) as an adjunct in individuals with T1DM and overweight or obesity on insulin pumps and poor glycemic control. This 26-week, placebo-controlled study included 100 participants with HbA1c > 8% and BMI > 25 kg/m². Liraglutide significantly reduced total insulin requirements by 8 units/day (16% reduction, *P* = 0.008) and led to an average weight loss of 6.8 kg. It did not significantly improve HbA1c compared to placebo, but increased time within the target glycemic range (4–10 mmol/L or 71–180 mg/dL). The risk of hypoglycemia remained unchanged between groups. Additionally, liraglutide delayed gastric emptying, although there were no significant differences in postprandial glucagon and GLP-1 levels between groups [23]. As mentioned earlier, GLP-1RAs are not FDA-approved for T1DM due to a lack of conclusive trials. However, according to a consensus opinion from the Diabetes Technology Society, GLP-1RAs may improve glycemic and metabolic outcomes without greatly increasing severe hypoglycemia or diabetic ketoacidosis (DKA) risk in adults with T1DM using automated insulin delivery (AID) systems, making this an area of future interest [24].

When focusing on body composition in T1DM with obesity, a 26-week randomized controlled trial evaluating liraglutide treatment found significant weight loss, primarily through a reduction in fat mass (3.4 ± 0.6 kg, *p* = 0.003), including a notable decrease in visceral fat according to Magnetic Resonance Imaging (MRI) quantification, with no impact on lean mass as measured by Dual-Energy X-ray Absorptiometry (DEXA). This reduction in fat mass accounted for about 82% of the total weight loss. Additionally, HbA1c decreased by 0.41 ± 0.18% (*P* = 0.001) from baseline, and by 0.29 ± 0.19% compared to placebo (*P* = 0.1). There was no increase in hypoglycemia, while time spent in normoglycemia increased (*P* = 0.015) and time in hyperglycemia decreased (*P* = 0.019) [25]. Another trial aimed at investigating the mechanisms behind the observed weight loss found no sustained changes in subcutaneous adipose tissue after 26 weeks of liraglutide treatment. These results highlight liraglutide’s potential in promoting weight loss, particularly in visceral fat, and suggest that further research is needed to explore its mechanistic effects [26].

Regarding the addition of liraglutide to other medical treatments, a recent retrospective analysis of electronic health records assessed the effects of liraglutide and dapagliflozin (SGLT2 inhibitor) on body weight and glycemic control in people with T1DM over 12 months. The study stratified participants, among other parameters, by weight (BMI < 30 and BMI > 30 kg/m^2^) and included four groups: control (*n* = 80), SGLT2i (*n* = 94), GLP1-RA (*n* = 82), and a combination of both drugs (combo, *n* = 40). The results showed that while both liraglutide and dapagliflozin individually improved body weight and HbA1c, the combination therapy yielded the most significant weight loss (9.0%, *p* < 0.001) and HbA1c reduction (0.6%, *p* < 0.001). Furthermore, the combo group showed greater improvements in lipid profiles compared to baseline, with no increased risk of DKA or hypoglycemia. These findings suggest that combining SGLT2 inhibitors and GLP-1RAs may provide superior benefits for weight loss and glycemic control in individuals with T1DM and obesity, without increasing severe adverse events [27].

Altogether, liraglutide added to insulin results in a modest HbA1c reduction (approximately 0.5%), and a body weight reduction of 5–6 kg, especially during the first three months of treatment [28]. Adverse events with liraglutide added to insulin include gastrointestinal issues and a higher risk of ketosis, particularly at higher doses (1.8 mg), highlighting the need for close monitoring and individualized dosing [28].

#### Semaglutide

A few studies have explored the effects of semaglutide on weight management and glycemic control in individuals with T1DM and overweight or obesity. A retrospective chart review of 50 patients who received semaglutide for one year, with the dose titrated from a mean of 0.63 mg at 3 months to 0.92 mg at 12 months, demonstrated significant improvements in body weight, BMI (mean 7.9% reduction), HbA1c, and continuous glucose monitoring (CGM) metrics, such as glucose variability and time in range (TIR). These findings suggest that semaglutide is effective in reducing body weight and enhancing glycemic control in real-world settings, but further well-structured clinical trials are needed for confirmation in this particular population [29].

The STEMT trial, another real-world study assessing the impact of weekly semaglutide (1 mg) on 20 adults with T1DM and obesity, further reproduced these results. Following six months, 60% of participants achieved at least a 5% weight loss, with a mean HbA1c reduction of 0.3%. Although insulin requirements decreased by 13.5%, some subjects (30%) did not experience HbA1c reductions. Semaglutide was well-tolerated, with gastrointestinal side effects leading to treatment discontinuation in only two participants [30].

A recent study by Grassi et al., involving eleven patients with T1DM using a lower dose of semaglutide (0.5 mg once weekly) showed promising outcomes, with an 11% weight loss (average of 8.8 kg). Participants also significantly reduced their carbohydrate intake, which correlated with decreased insulin requirements. Total daily insulin (TDD) dropped from an average of 45.6 to 38.5 U/day, reflecting the proportional decrease in both basal and bolus insulin [31].

Taken together, these findings indicate that semaglutide is a promising therapeutic option for improving weight management and metabolic control in T1DM patients with obesity or overweight, though further large-scale, randomized clinical trials are necessary to fully assess its long-term efficacy and safety.

#### Tirzepatide

Tirzepatide, a once-weekly dual agonist targeting both GLP-1 and GIP receptors, has demonstrated significant efficacy in managing both glycemic control and weight in individuals with type 2 diabetes (T2DM). However, its use in T1DM remains off-label, with limited studies exploring its impact on this population [32].

A retrospective observational study of 26 adults with T1DM reported notable improvements in glycemic control, with reductions in HbA1c of 0.45% (at 3 months) and 0.59% (at 8 months). Body weight decreased significantly by 3.4% (at 3 months), 10.5% (at 6 months), and 10.1% (at 8 months). TIR increased by a substantial 12.6%, and time in tight target range (TITR) increased by 10.7% at 3 months, while time above range (TAR) decreased by 12.6%, all significantly. These improvements were sustained over 8 months. Although tirzepatide was generally well-tolerated, two patients discontinued treatment due to severe hypoglycemia (*n* = 1) and severe constipation (*n* = 1), with no incidents of DKA [33].

In a separate retrospective study, adults with T1DM and obesity who received tirzepatide for at least 3 months experienced a median weight loss of 8% (range 5–14), a mean reduction in HbA1c by 1%, and a 32% (range 6–45) decrease in Total Daily Dose (TDD). TIR increased by 29%, and TAR decreased by 28%, with a trend toward a reduction in time below range (TBR) by 32%. Although side effects were reported in 26% of patients, with nausea being the most common (15%), no severe hypoglycemia or DKA occurred [34]. These findings suggest that tirzepatide could offer substantial benefits in weight management and glycemic control for individuals with T1DM, but, as pointed out earlier with semaglutide, further investigation is needed (See Table 2).

**Table 2:** Summary of outcomes of studies investigating the use of GLP-1 RA in T1DM. ETD: estimated treatment difference, NS: not specified, tdd = total daily dose

Study	Type of study	Duration	Medication-dose	Outcome
[18]	Network meta-analysis of RCTs	30.8 ± 14.5 w	10µg exenatide VS placebo (insulin alone)	BW change − 5.1kg (-8.4, -2.0) No change of HbA1c, TDD, hypoglycemic events, VS insulin
[18]	Network meta-analysis of RCTs	30.8 ± 14.5 w	1.2mg liraglutide (3 studies), 1.8mg (1 study) VS placebo (insulin alone)	No change of BW, HbA1c, TDD, hypoglycemic events, VS insulin
[19]	Randomized, double-blind, placebo-controlled trial (Lira-1)	24 w	1.8 mg liraglutide (maintenance dose) VS placebo	↓ incident rate ratio of hypoglycemic events: 0.82 (0.74 to 0.90), ↓ -5.8 IU (-10.7 to -0.8) of bolus insulin dose, ↓ -6.8 kg (-12.2 to -1.4) of BW, ↑ 7.5 bpm (2.8 to 12.2) of heart rate, ↓ -0.6 (-1.1 to -0.07) perceived hypoglycemia, no sign. changes in HbA1c and glycemic variability, 58% nausea VS 10% placebo
[20]	Double-blind, treat-to-target randomized trial	52 w	liraglutide (1.8, 1.2, or 0.6 mg) VS placebo (insulin alone)	HbA1c change: ETD: -0.20% (-0.32; -0.07) (1.8 mg) ETD: -0.15% (-0.27; -0.03) (1.2 mg) ETD: -0.09% (-0.21; 0.03) (0.6 mg) Insulin dose (1.8, 1.2, 0.6mg): ↓ Estimated ratio: 0.92 (0.88; 0.96) ↓ Estimated ratio: 0.95 (0.91; 0.99) No change (1.00; 0.96 to 1.04) BW change (1.8, 1.2, 0.6mg): ↓ -4.9 kg (-5.7; -4.2) ↓ -3.6 kg (-4.3; -2.8) ↓ -2.2 kg (-2.9; -1.5) Symptomatic hypoglycemia (1.8, 1.2, 0.6mg): ↑ Rate ratio: 1.31 (1.07; 1.59) ↑ Rate ratio: 1.27 (1.03; 1.55) ↑ Rate ratio: 1.17 (0.97; 1.43) Hyperglycemia with ketosis (1.8, 1.2, 0.6mg): ↑ Event rate ratio: 2.22 (1.13; 4.34) No significant change No significant change
[21]	Randomized Placebo-Controlled Clinical Trial	12 w	0.6, 1.2, and 1.8 mg liraglutide	HbA1c change (1.8, 1.2, 0.6mg): no sign. change, -0.78 ± 0.15% (-8.5 ± 1.6 mmol/mol) (P < 0.01), no sign. change, Glycemic Variability (1.8, 1.2, 0.6mg): No sign. change, ↓ 5 ± 1% (P < 0.01), No sign. change, ↓ TDD with 1.2 and 1.8mg (P < 0.05), BW change (1.8, 1.2, 0.6mg): -5 ± 1 kg (P < 0.05), -5 ± 1 kg (P < 0.05),-2.7 ± 0.6 kg (P < 0.01), no sign. increases in gastrointestinal adverse events
[22]	Retrospective analysis	180 ± 14 d	1.8mg liraglutide (maintenance dose)	Mean glucose (mg/dL)↓ 191 ± 6 → 170 ± 6, P =.002 HbA1c (%) ↓ 7.89 ± 0.13 → 7.46 ± 0.13, P =.001 Body weight (kg) ↓ 96.20 ± 3.68 → 91.56 ± 3.78, P <.0001 Total insulin (U/day) ↓ 73 ± 6 → 60 ± 4, P =.008 Bolus insulin (U/day) ↓ 40 ± 5 → 29 ± 3, P =.011 Systolic BP (mmHg) ↓ 130 ± 3 → 120 ± 4, P =.020 No increase in hypoglycemia
[23]	Randomized, double-blinded, placebo-controlled trial (The Lira Pump trial)	26 w	1.8 mg liraglutide VS placebo, added to CSII treatment	HbA1c (%) change: ETD: 0.7(− 1.0;−0.3), Weight (kg) change: ETD: −6.3(− 8.2;−4.5), TDD change (IU/day): ETD: −7.7(− 13.5;−2), CGM target(%) change: EDT: 9.4(0.2;18.5), no difference in hypoglycemia risk
[25]	RCT	26 w	1.8 mg/d liraglutide	↓ fat mass (3.4 ± 0.6 kg, p = 0.003), HbA1c ↓0.41 ± 0.18% (P = 0.001), TIR ↑ 7 ± 2 (from 42 ± 3% to 49 ± 2%) (P = 0.015), TAR ↓8 ± 3 (from 52 ± 4% to 44 ± 4%) (P = 0.019), no increase in hypoglycemia, from baseline
[27]	Retrospective analysis	12 mo	Liraglutide (NS), VS SGLT-2i VS combo	Weight change (GLP1-RA Group, Combo Group): ↓ 8.2% (8.5%), ↓9.0% (8.4%), P < 0.001, HbA1c reduction: ↓ 0.3% (0.7%), ↓ 0.6% (0.8%), P < 0.001, no increased risk of DKA or hypoglycemia
[29]	Retrospective analysis	12 mo	Semaglutide 0.63 mg at 3 mo to 0.92 mg at 12 mo	BMI change (% ± SD): ↓ 7.9% ± 2.6% VS no sign. in placebo, HbA1c change (% ± SD): ↓ 0.66% ± 0.15% VS 0.4% ± 0.13%, TIR ↑ 7.4% ± 2.9% VS ↓ 0.7% ± 3.2% at 9 mo
[30]	Real-world exploratory study (STEMT trial)	NS	1.0 mg semaglutide once-weekly	Mean body weight change (kg, Mean ± SD): -8.5 ± 7.8 (range: +1.5 to -24.7), Mean HbA1c reduction (% ± SD): 0.3 ± 0.7, Subjects achieving ≥ 5% weight loss (%)= 60%, Subjects achieving ≥ 10% weight loss (%)= 40%
[33]	Single-center, retrospective, observational study	8 mo	≥ 2.5 mg tirzepatide (up titrated by physician)	HbA1c (%) change: −0.59 (− 1.29, 0.10), Mean sensor glucose (mg/dL) change: −16.9 (− 28.4, − 5.3), TIR (%) change: +11.5 (+ 4.1, + 18.8), TAR (%) change: −11.4 (− 18.8, − 3.9), TBR (%) change: −0.1 (− 1.2, + 1.0), Percent change in BMI (kg/m^2^): −9.9 (− 18.4, − 1.4), % change in insulin TDD per day (IU/day): −24.3 (− 38.9, − 9.7), at 8 mo from baseline
[34]	Retrospective study	3–16 mo	≥ 2.5 mg tirzepatide	BW change: -8.5% (Q1-Q3: 5.3%-13.8%), P <.01, HA1c change: 0.9% (Q1-Q3: 0.3%-1.1%), P <.0001, insulin TDD change: -31.6% (Q1-Q3: -48.0% to -10.9%), P <.01, from baseline, no increased incidence of hypoglycemia, Nausea (13.7%)

### Bariatric Surgery (BS)

Bariatric surgery is a highly effective and efficient intervention for managing obesity in the long term. It is recommended for individuals with a BMI > 35 kg/m^2^, regardless of presence, absence, or severity of co-morbidities and should be considered for individuals with metabolic disease and a BMI of 30–34.9 kg/m^2^ [35]. This surgical approach offers more than just weight reduction; it substantially improves metabolic health by reducing lipotoxicity in the liver and skeletal muscles and attenuating the proinflammatory environment associated with obesity, an effect which is linked to decreased insulin resistance. Furthermore, Roux-en-Y gastric bypass (RYGB) predominantly promotes the secretion of incretin and anorexigenic hormones such as GLP-1 and peptide YY (PYY) and induces changes in intestinal microbiota, bile acids, and brain plasticity, contributing, among others, to better glucose metabolism [36].

BS has demonstrated beneficial effects on metabolic syndrome components, including hyperglycemia, dyslipidemia, hypertension, and inflammation [37]. However, its role in managing T1DM remains under investigation. Despite improvements in weight and metabolic markers, achieving optimal glycemic control in T1DM patients undergoing BS remains challenging [37].

A study investigating clinical outcomes in T1DM patients who underwent BS revealed significant weight and BMI reductions [38]. Six months post-surgery, patients experienced substantial weight loss, with BMI decreasing from a mean of 39.5 kg/m² to 27.5 kg/m² after two years, a total reduction of 12 units. Although BMI slightly increased in the third year, the mean BMI at follow-up was still 9 units lower than pre-surgery levels. In contrast, patients with T1DM who did not undergo surgery experienced a modest BMI increase over the same period. Glycemic improvements were also noted, with a 0.6% reduction in HbA1c during the first six months post-surgery, likely due to strict caloric restriction. However, HbA1c levels returned to baseline after one year and remained comparable to those in the non-surgical cohort throughout follow-up [38]. The underlying reasons for this are not well comprehended; however, authors suggest that potential challenges in long-term behavioral adherence may play a role [38].

A systematic review and meta-analysis of 28 studies confirmed that BS leads to significant reductions in biochemical markers of diabetes status among T1DM patients with severe obesity [39]. Notable improvements included lower blood pressure, triglycerides, and cholesterol levels, as well as a dramatic decrease in insulin requirements by approximately 50%, with minimal heterogeneity when lower-quality studies were excluded. The reduction in HbA1c levels, averaging 0.93%, was modest but consistent [39]. These findings align with another analysis, where the weighted mean differences for insulin use and HbA1c were − 10.59 units/day and − 0.71, respectively, alongside a notable reduction in BMI from 42.6 ± 4.7 kg/m² preoperatively to 29.4 ± 4.7 kg/m² postoperatively. Roux-en-Y gastric bypass (RYGB) was the most frequently implemented procedure, performed in 72.5% of cases [36]. In another meta-analysis conducted in 2022, which included 30 studies and 706 patients, Roux-en-Y gastric bypass (RYGB) was once again identified as the most frequently performed procedure (70.4%) compared to sleeve gastrectomy (18.6%), mirroring findings from other research conducted in the same year [36, 39]. The authors proposed that the observed reductions in insulin requirements were driven by improved β-cell function, preservation of remaining β-cell mass, and enhanced hepatic insulin sensitivity [40]. However, significant HbA1c reductions were not consistently observed, emphasizing the variable impact of BS on long-term glycemic control [40].

While BS is generally safe and effective, its application in T1DM patients presents unique challenges. Postoperative complications include DKA and severe hypoglycemia, both of which require careful management due to altered glucose metabolism after surgery. Hypoglycemia incidence can reach 70% in the postoperative period, necessitating accurate insulin titration and continuous glucose monitoring [41]. DKA has been reported in 15–25% of patients, with rates influenced by perioperative factors such as anesthesia, surgical stress, inadequate insulin management, postoperative infections, and dehydration [36, 38, 40]. Proper insulin adjustments are essential to prevent these complications [36, 40]. Other short-term complications include ulcers, incisional hernias, esophageal dysmotility, persistent nausea, and nutritional deficiencies. The reported surgical complication rates vary between 4% and 25%, depending on the procedure performed. Despite these risks, no excess mortality has been observed among T1DM patients undergoing BS [42].

BS may provide significant benefits in T1DM patients with obesity, including substantial weight loss, reduced insulin requirements, and metabolic improvements. However, its impact on glycemic control remains inconsistent, and the procedure is associated with risks such as DKA and hypoglycemia, which require careful postoperative management. These limitations highlight the importance of an individualized approach when considering BS for this population, including personalized monitoring and adjustments. Additional research is required to thoroughly comprehend the long-term outcomes and improve the management strategies for T1DM patients undergoing BS [37, 42].

### Limitations and Future Directions

Although the understanding of the gut-brain axis has advanced significantly in recent years, several limitations exist in the current body of literature specific to T1DM and obesity. Most studies are either extrapolated from type 2 diabetes populations, which may not fully capture the autoimmune and metabolic complexities of T1DM. Additionally, heterogeneity in treatment regimens, especially insulin dosing and its impact on appetite regulation, poses a challenge in study standardization. Regarding long-term cardiovascular or renal outcomes evidence is lacking, as patients with T1D were excluded from major trials. Furthermore, the potential of early initiation of GLP-1RAs to preserve β-cell function is still under investigation. Future studies are needed to clarify these aspects and to ensure safe and effective integration into T1D management.

## Conclusion

The rising prevalence of obesity among individuals with T1DM presents a significant clinical challenge due to its impact on glycemic control, insulin resistance, and cardiometabolic risk. The interplay between T1DM, obesity, and the gut-brain axis underscores the complex hormonal and neural mechanisms involved in energy balance, food intake regulation, and metabolic health. Dysregulation of key gut hormones, including GLP-1, GIP, and amylin, may contribute to weight gain and complicate diabetes management. Lifestyle modifications, GLP-1 RAs based pharmacotherapy and BS have emerged as potential strategies to address obesity in this population. While early evidence suggests promising metabolic and glycemic benefits, concerns regarding safety and the need for longer follow-up persist. Further research is essential to elucidate the precise mechanisms linking the gut-brain axis to weight regulation in T1DM and to establish the efficacy, safety, and long-term outcomes of these interventions.

## Key References

- Vilsbøll T, Krarup T, Sonne J, Madsbad S, Vølund A, Juul AG, et al. Incretin secretion in relation to meal size and body weight in healthy subjects and people with type 1 and type 2 diabetes mellitus. J Clin Endocrinol Metab. 2003;88:2706–13.
-
  - Decreased GLP-1 secretion may impair insulin release in type 2 diabetes, while beta-cell glucose sensitivity can be modulated by meal size.
- Dejgaard TF, Frandsen CS, Hansen TS, Almdal T, Urhammer S, Pedersen-Bjergaard U, et al. Efficacy and safety of liraglutide for overweight adult patients with type 1 diabetes and insufficient glycaemic control (Lira-1): a randomised, double-blind, placebo-controlled trial. Lancet Diabetes Endocrinol. 2016;4:221–32.
-
  - GLP-1 receptor agonists may improve glycemic control and metabolic outcomes in adults with type 1 diabetes.
- Dejgaard TF, Schmidt S, Frandsen CS, Vistisen D, Madsbad S, Andersen HU, et al. Liraglutide reduces hyperglycaemia and body weight in overweight, dysregulated insulin-pump-treated patients with type 1 diabetes: The Lira Pump trial-a randomized, double-blinded, placebo-controlled trial. Diabetes Obes Metab [Internet]. 2020;22:492–500. Available from: https://doi.org/10.1111/dom.13911
-
  - Liraglutide reduced HbA1c, insulin requirements, and body weight without raising hypoglycemia risk in CSII-treated adults with T1DM and suboptimal glycemic control.
- Shah VN, Peters AL, Umpierrez GE, Sherr JL, Akturk HK, Aleppo G, et al. Consensus Report on Glucagon-Like Peptide-1 Receptor Agonists as Adjunctive Treatment for Individuals With Type 1 Diabetes Using an Automated Insulin Delivery System. J Diabetes Sci Technol. 2025;19:191–216.
-
  - GLP-1RAs may serve as effective adjuncts to AID in adults with T1DM, improving glycemic and metabolic outcomes without increasing the risk of severe hypoglycemia or DKA.

## Data Availability

No datasets were generated or analysed during the current study.

## Abbreviations

AID: Automated Insulin Delivery
BMI: Body Mass Index
BS: Bariatric Surgery
CGM: Continuous Glucose Monitoring
DKA: Diabetic Ketoacidosis
DEXA: Dual-Energy X-ray Absorptiometry
FDA: Food and Drug Administration
GIP: Glucose-dependent Insulinotropic Polypeptide
GLP-1: Glucagon-Like Peptide-1
GLP-1RAs: Glucagon-Like Peptide-1 Receptor Agonists
IGFBP-1: Insulin-Like Growth Factor Binding Protein-1
MRI: Magnetic Resonance Imaging
PYY: Peptide YY
RYGB: Roux-en-Y Gastric Bypass
SGLT2i: Sodium-Glucose Cotransporter-2 Inhibitor
TAR: Time Above Range
TBR: Time Below Range
TDD: Total Daily Dose
T1DM: Type 1 Diabetes Mellitus
TIR: Time in Range
TITR: Time in Tight Target Range
